# Insights into molecular mechanisms of poxvirus evolution and host range through studies of poxvirus inhibitors of protein kinase R

**DOI:** 10.1128/jvi.01768-24

**Published:** 2026-09-25

**Authors:** Stefan Rothenburg, Dewi Megawati, Ryan Bruneau, Loubna Tazi

**Affiliations:** 1 Department of Medical Microbiology and Immunology, School of Medicine, University of California Davis 8789, Davis, California, USA; 2 Department of Microbiology and Parasitology, Faculty of Medicine and Health Sciences, Warmadewa University 202244 , Bali, Indonesia; 3 Biotechnology and Bioengineering, Sandia National Laboratories 111651, Livermore, California, USA; New York University Department of Microbiology, New York, New York, USA

**Keywords:** poxvirus, PKR, decapping enzyme, E3L, K3L, NF-kB, bunny paradox, CREP, GADD34, host range

## Abstract

Protein kinase R (PKR) acts both as a sensor for virus infections, by detecting double-stranded (ds) RNA, and as an antiviral effector by phosphorylating the alpha subunit of eukaryotic translation initiation factor 2 (eIF2). Consequently, viruses have evolved many mechanisms to evade this potent antiviral protein, which in turn led to the fast evolution of PKR. There is an emerging understanding that these molecular arms races have important implications for the virulence and host range of viruses, which was in part propelled by research on PKR and poxvirus PKR inhibitors. At least four direct PKR pathway inhibitors, which either prevent PKR activation, inhibit interaction with eIF2α, or reverse eIF2α phosphorylation, evolved independently in different poxvirus clades, while poxvirus decapping enzymes provide another mechanism to subdue PKR activation via the reduction of dsRNA formation. In addition to discussing the functions and distributions of poxvirus PKR pathway inhibitors, we focus on host species-specific PKR-inhibitor interactions and their consequences for virus host range and virulence, including differential NF-κB pathway activation. We further highlight how research with PKR inhibitor-deficient poxviruses led to seminal discoveries elucidating molecular mechanisms for virus evolution, including gene amplification and horizontal gene transfer.

## INTRODUCTION

**Fig 1:**
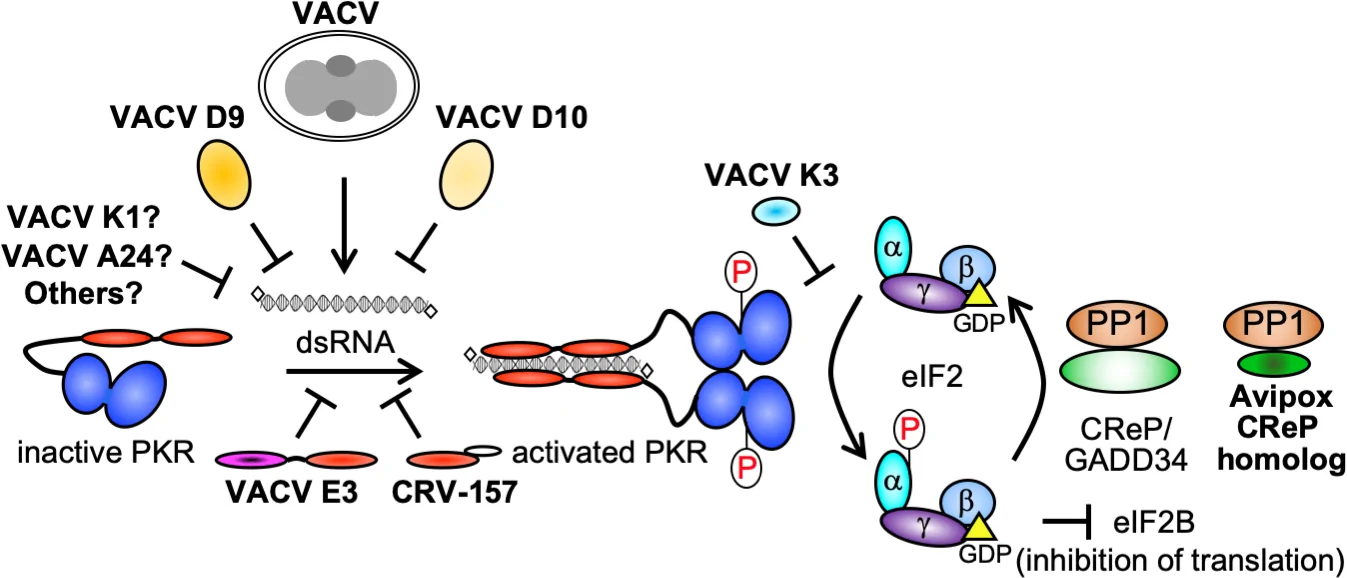
Inhibition of the PKR pathway by poxviruses. Host proteins and complexes are labeled with regular fonts and are shaded lightly in the center. Poxvirus proteins are labeled with bold letters and are shaded darkly in the center. Related protein domains are indicated by similar colors. Phosphorylated proteins are marked by a circled P. CReP, constitutive repressor of eIF2α phosphorylation; CRV, crocodilepox virus; ds, double-stranded; eIF2, eukaryotic translation initiation factor 2; eIF2B, eukaryotic initiation factor 2B; GADD34, growth arrest and DNA damage-inducible protein 34; GDP, guanosine diphosphate; PP1, protein phosphatase 1; PKR, protein kinase R; VACV, vaccinia virus.

Pattern recognition receptors (PRRs) recognize molecular patterns shared by multiple pathogens and constitute a first line of defense against invading pathogens. One of the first identified PRRs is protein kinase R (PKR/EIF2AK2) (1–6). Mammalian PKRs contain two N-terminal dsRNA-binding domains (dsRBDs) and a C-terminal kinase domain, which are connected by a linker (6, 7). PKR recognizes cytosolic double-stranded (ds) RNA, which is formed during the replication cycle of most viruses, and subsequently induces PKR dimerization. Dimerized PKR undergoes autophosphorylation and subsequently phosphorylates the alpha subunit of eukaryotic translation initiation factor 2 (eIF2). eIF2 is required for canonical, cap-dependent translation initiation. In order to efficiently translate their RNAs, viruses evolved diverse strategies to add 5′-methyl-GTP caps by either using their own or cellular capping enzymes or by snatching cap structures from cellular mRNAs (8). Phosphorylated eIF2 acts as an inhibitor of eukaryotic initiation factor 2B (eIF2B), which normally functions as a guanine nucleotide exchange factor, exchanging guanosine diphosphate (GDP) for guanosine triphosphate (GTP) on eIF2, an essential step in translation initiation (Fig. 1). Because of the inhibition of eIF2B, eIF2-dependent translation is strongly inhibited, which affects both cellular and viral transcripts (9). Another consequence of eIF2α phosphorylation is that proteins with shorter half-lives are depleted faster than more stable proteins, which can lead to the modulation of signaling pathways, such as the immune-regulatory NF-κB pathway, which is activated through the rapid turnover of its inhibitor IκB, following eIF2α phosphorylation (10–14). Together, the effects of eIF2α phosphorylation result in potent antiviral activity. In mammals, dephosphorylation of eIF2α is mediated by two proteins: constitutive repressor of eIF2α phosphorylation (CReP/PPP1R15B) and growth arrest and DNA damage-inducible protein 34 (GADD34/PPP1R15A), both of which interact with protein phosphatase 1 (PP1) and eIF2 to mediate eIF2α dephosphorylation and thus reverse the effect of PKR and other eIF2α kinases (Fig. 1) (reviewed in reference 15). While it is thought that most of the effects of PKR are eIF2α phosphorylation-dependent, eIF2-independent effects of PKR have also been described, presumably mediated by the interaction of PKR with other PRRs such as MDA5 (16).

In addition to PKR, three other eIF2α kinases are found in mammals. They all contain an eIF2α kinase domain but are activated by other stressors via different regulatory domains: heme-regulated inhibitor (HRI, EIF2AK1) is activated by metabolic and environmental stress; PKR-like endoplasmic reticulum kinase (PERK, EIF2AK3) is activated by endoplasmic reticulum stress and is a component of the unfolded protein response; and general control nonderepressible 2 (GCN2, EIF2AK4) is activated after amino acid starvation (reviewed in reference 15). These eIF2α kinases have also been implicated in the host response to viruses (reviewed in references 17–19).

Here, we review the diverse ways poxviruses evolved inhibitors of the PKR pathway and how studies of PKR and PKR inhibitors from poxviruses have led to seminal discoveries resulting in a better understanding of the molecular basis of host-virus interactions, virus-host range, and host-virus evolution.

## PKR DIVERSIFICATION

The potent antiviral effects of PKR exerted strong selective pressure on virus evolution and have resulted in many viruses evolving inhibitors of the PKR pathway (20–22). As a consequence, PKR is one of the fastest evolving vertebrate genes. Positively selected sites in PKR have been identified in multiple PKR data sets from various vertebrates, primates, bats, rodents, and ruminants (23–27). Rapid PKR diversification is likely aided and punctuated by multiple gene duplication events, first described in various fish and amphibian species and more recently in some bat species (25, 28). In all of these cases, either two or three PKR copies are tandemly arranged, possibly providing increased resistance to viruses. Furthermore, some PKRs encode up to three dsRBDs, possibly representing another adaptation. Finally, some fish species contain a PKR-related eIF2α kinase, called PKZ, which contains two Z-nucleic acid domains in place of the dsRBDs and is tandemly arranged with a PKR gene containing three dsRBDs in zebrafish (28, 29). PKZ likely increases the range of viral nucleotide sequences detected during infection and demonstrates adaptive flexibility in the evolution of eIF2α kinases.

## POXVIRUSES

Poxviruses are large dsDNA viruses, ranging in genome size between 122 kb in Cetacean poxvirus 1 (30) and 460 kb in Carp edema virus (31), and contain up to 392 open reading frames. The *Poxviridae* family is grouped into two subfamilies, *Chordopoxvirinae*, which infect vertebrates and for which 18 genera are currently recognized, and the insect-infecting *Entomopoxvirinae*, which contains four genera (32).

Expression of poxvirus genes is temporally regulated by stage-specific poxvirus promoters, leading to early, intermediate, and late expression patterns, with early transcription starting as early as virions enter a host cell (reviewed in reference 33). Poxviruses contain inverted terminal repeat (ITR) regions at genome termini that can be longer than 20 kb, which results in the duplication of genes that are present in this region. Whereas the ITR regions are relatively stable during virus replication, they can expand or retract under evolutionary pressures, leading to alterations in the expression levels of affected genes (34). Poxvirus gene nomenclature is not straightforward and varies from one poxvirus to another. Traditionally, poxvirus genes were named according to their location on restriction enzyme fragments, which were organized by size and labeled by letters, the relative position within one fragment labeled by a number, and the direction of transcription, either R (right, upper strand) or L (left, lower strand), e.g., vaccinia virus (VACV) E3L, for being the third gene on the E fragment being transcribed into the left direction. For encoded protein names, the orientation is omitted, e.g., E3. Because other viruses, such as variola and monkeypox viruses, yielded different restriction fragments, the names of orthologous genes often do not match the VACV names. In addition, proteins were often given individual, sometimes virus-specific names. Alternatively, open reading frames (ORFs) in more recently sequenced genomes were annotated according to the ORF position in the genome starting from the left side. A key problem with both approaches is that orthologous genes and proteins in other poxviruses cannot easily be identified. In an attempt to overcome this confusion, Senkevich et al. proposed a unified gene nomenclature for orthopoxvirus genes (OPGs), which was based on thorough comparative genome analyses, e.g., E3L is represented by OPG065 (35). The OPG nomenclature is very useful and can also be applied to viruses from other genera. However, it is complicated by different evolutionary trajectories among poxvirus genera. For example, some poxviruses have multiple homologs of a given protein, which often evolved through lineage-specific duplications or recombination with other poxviruses. In addition, many poxvirus lineages contain genes that do not have homologs in orthopoxviruses. For this review, we use the gene and protein names used for the viruses in which the initial discoveries were made and indicate OPG names when applicable (Table 1). For orthologs from non-orthopoxviruses, we also indicate the currently used names.

**TABLE 1:** Orthopoxvirus genes discussed in this review

VACV-Copenhagen gene name	OPG name
A24R	151
A35R	163
A7L	133
C7L	027
D1R	113
D5R	117
D9R	121
D10R	122
E3L	065
E9L	071
F4L	048
I8R	084
J6R	105
K1L	039
K3L	041

During the poxvirus replication cycle, large amounts of dsRNA are formed as a result of overlapping, converging transcription of neighboring genes that are transcribed from the upper and lower strand of the genome, respectively (36–39). This dsRNA can be sensed by a group of PRRs that include PKR, oligoadenylate synthetase (OAS), retinoic acid-inducible gene I (RIG-I), melanoma differentiation-associated protein 5 (MDA5), and toll-like receptor 3 (TLR3) and induce a diverse set of antiviral pathways, which are targeted by several poxvirus proteins (40).

The host range of individual poxviruses can vary greatly even within the same genus. For example, the orthopoxvirus genus includes viruses that are restricted to one host, such as variola virus, the causative agent of smallpox, which only infects humans, and cowpox and monkeypox viruses, which productively infect multiple and diverse sets of hosts (41, 42). Because poxviruses use ubiquitous receptors such as glycosaminoglycans to enter host cells (43), the species-specificity of poxviruses is determined at post-entry steps, which include the efficient inhibition of both innate and adaptive host immune responses. The molecular determinants of poxvirus host range are only partially understood. A group of poxvirus genes has been identified as host range genes, which through experimental infection assays have been found to be necessary or important for productive infection in a subset of cell lines, while dispensable for the infection of others (41, 44). The cell line tropism of host range gene-deleted viruses often correlates with the species that they originated from. This is discussed in detail for two genes from VACV, called E3L (OPG065) and K3L (OPG041) in the Copenhagen strain, in the respective sections below.

## INHIBITION OF PKR BY VACV E3

VACV E3 is a dsRNA binding protein that was initially identified by its ability to bind to the synthetic dsRNA analog polyinosinic-polycytidylic acid (poly I:C) and was shown to inhibit the activation of PKR (45–47). E3 contains a C-terminal dsRBD and an N-terminal Z-nucleic acid (NA)-binding domain (Zα) (48–50). E3L encodes both a full-length (p25) isoform and an N-terminally truncated isoform (p20) lacking a functional Zα domain, due to the use of an internal start codon. E3L expression is controlled by a strong early promoter and therefore can inhibit PKR early during infection (51). Inhibition of PKR is dependent on the presence of the dsRBDs, and mutation of key residues in E3 that contribute to the binding of dsRNA greatly reduced its ability to inhibit PKR and caused severe replication defects in HeLa cells of VACV carrying these mutations (52, 53). Moreover, a genetically modified VACV that contained a deletion of E3L (VACV∆E3L) or a truncation of the 26 C-terminal amino acids (aa) was strongly attenuated in a mouse infection model (54). In addition to PKR, E3 has been described as an inhibitor of other PRRs, including OASs, Z-DNA binding protein 1, RIG-I/MDA5, TLR8, TLR9, and RNA polymerase III (reviewed in reference 40). In respect to VACV replication, PKR and OAS/RNase L seem to be the most important targets of E3, demonstrated by complete rescue of E3L-deleted VACV in PKR-depleted or deficient HeLa, HAP1, and 293 cells (25, 55, 56). In A549 cells, inactivation of both PKR and RNase L was sufficient to rescue VACV∆E3L replication (56). Moreover, replication of a VACV strain deficient in both E3L and K3L (which encodes the unrelated PKR inhibitor K3) could be completely rescued by K3L orthologs from other poxviruses that were efficient inhibitors of the respective PKRs in the following cell lines: HeLa, gibbonTert-GF, African green monkey BSC-40, Pro1190, and CV-1, sheep OA1 and OA3, goat CSM, cow BT, pig PK15, mouse 3T3, and rabbit RK13 cells (25, 57–60).

Two models for how E3 inhibits PKR have been proposed. (i) According to the sequestration model, E3 binds and masks all dsRNA and thus precludes PKR from interacting with dsRNA (61, 62). (ii) The heterodimer model proposes that E3 forms heterodimers with PKR and thus prevents PKR homodimerization and activation (63, 64). The recent discovery of PKRs being resistant to E3 inhibition gives support to the heterodimerization model, although it does not exclude that sequestration of dsRNA might still be a factor (26).

## E3 ORTHOLOGS IN OTHER POXVIRUSES

**Fig 2:**
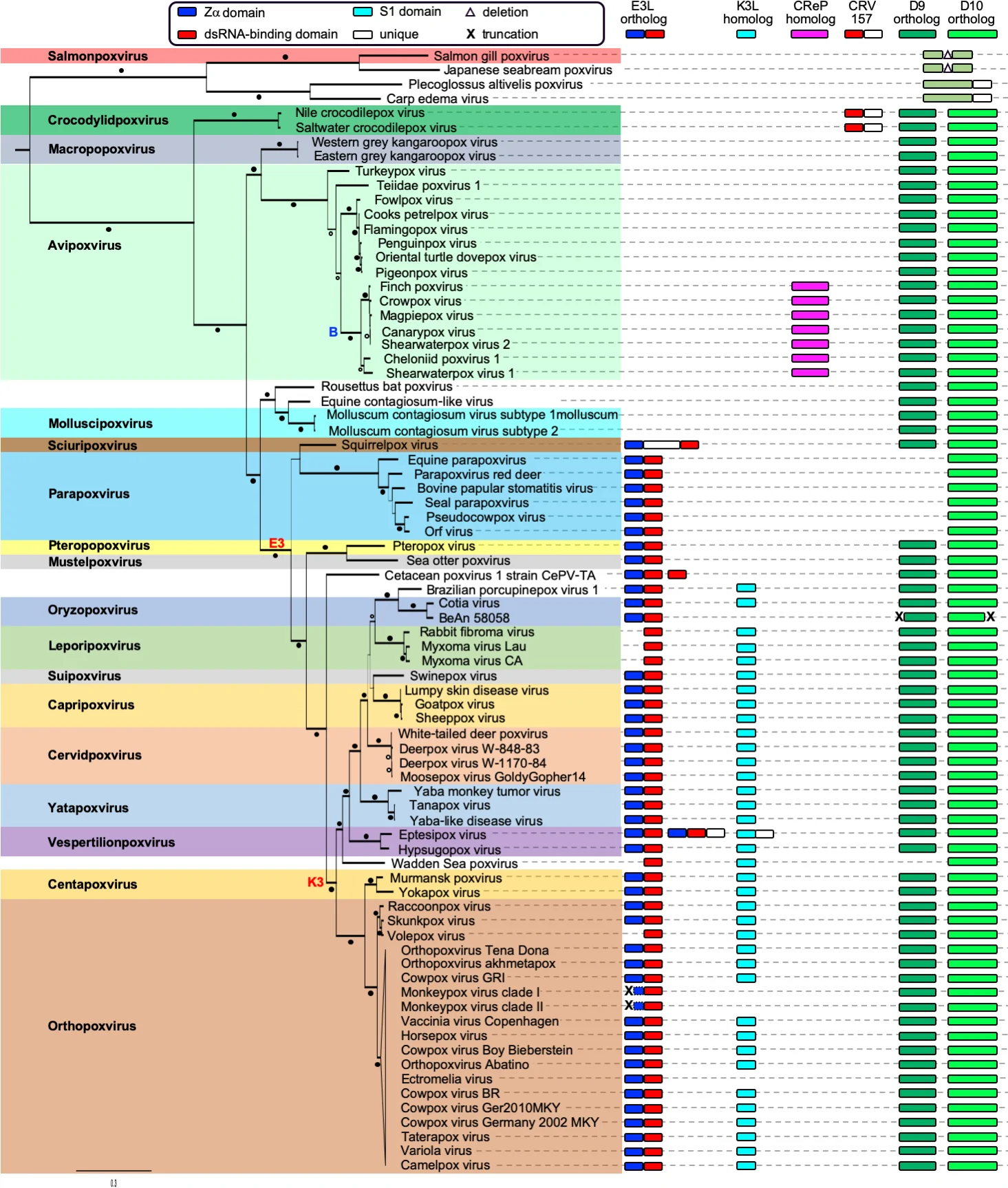
Presence of PKR pathway inhibitors in poxviruses. A poxvirus phylogeny was generated from sequences of seven conserved poxvirus proteins (E9-D5-J6-I8-A24-A7-D1) from 85 poxviruses. Individual proteins were aligned using MUSCLE (70). Prior to concatenation, poorly aligned fragments were removed using Gblocks 0.91b (71). A maximum likelihood phylogenetic tree was generated using 100 bootstrap replicates, as implemented in PhyML 3.0 (72), and visualized in FigTree v1.4.3 (http://tree.bio.ed.ac.uk/software/figtree/). The resulting phylogenetic tree was rooted to entomopoxviruses, which are not shown here for better visualization. Bootstrap support on tree branches is indicated by closed circles (≥95) or open circles (≥90). The presence of PKR pathway inhibitors, as determined using blastp and tblastn searches (https://blast.ncbi.nlm.nih.gov/Blast.cgi), is projected next to the branches. The branches of Old World orthopoxviruses were collapsed and shown as a triangle for better clarity. E3 and K3 indicate clades in which E3 and K3 homologs are found. Clade B avipoxviruses are indicated by a “B.”

To analyze the presence and domain organization of E3 orthologs in other poxviruses, we extended previous analyses and performed database searches and sequence comparisons with currently available sequences (65, 66). Consistent with this earlier work, E3 likely evolved in an ancestral chordopoxvirus after the split from the molluscipoxvirus-containing clade, labeled as the E3 clade in Fig. 2. Most of the viruses in the E3 clade have the same domain organization as VACV E3. Of note is that the Zα domain was independently lost in some viruses or clades, i.e., in leporipoxviruses, Wadden Sea poxvirus, volepox virus, and monkeypox virus (65–67). While the former viruses completely lost the Zα domain, monkeypox virus contains point mutations in the start codon and small deletions in the 5′ region of the ORF, and utilizes the second start codon corresponding to the shorter p20 isoform, which does not contain a functional Zα domain. The squirrelpox virus E3 ortholog contains a unique, highly acidic 180-aa long insertion between Zα and dsRBD. Whereas E3 homologs are single copy in most poxviruses, two poxviruses contain two copies. Cetacean poxvirus possesses two closely related, tandemly arranged copies (ORFs 20 and 21) that show 47% aa identity and probably originated from a tandem duplication (30, 68). While ORF 21 encodes both a Zα and dsRBD, ORF 20 contains only the dsRBD. The other virus is the bat-infecting eptesipox virus, which contains two divergent E3 homologs (ORFs 037 and 163) that show only 22% aa identity and which are located at distinct genomic locations (69). ORF 037 shows conserved synteny with all other E3L homologs, except for the only hypsugopox virus E3 homolog ORF 169, which shows synteny with eptesipox virus ORF 163. An ancestral vespertilionpoxvirus likely obtained a second copy from another poxvirus, and while the first E3 homolog was maintained in eptesipox virus, it was lost in hypsugopox virus. Eptesipox virus ORF 163 contains a unique 80-aa long extension at the C-terminus (68).

Work on E3 orthologs from other poxviruses has yielded interesting insights into conserved and divergent functions. A modified VACV containing the orf virus E3L ortholog OV20.0, which shares 31% identity on the protein level, in place of E3L, showed comparable effects as E3L in a plaque reduction assay, in which both orthologs mediated resistance against the antiviral effects of rabbit interferon-β in rabbit RK13 cells. This indicated that either E3 ortholog could overcome the interferon effects, which likely included elevated PKR expression. However, this virus was non-virulent after intranasal infection in mice, and virus replication was restricted to nose tissue (73). Like VACV E3L, OV20.0 also encodes two isoforms, which were both able to bind dsRNA, interact with PKR, and inhibit the activity of human PKR. Recombinant orf viruses expressing either full length or only the shorter isoform replicated equally well in goat fibroblasts. However, the virus expressing the shorter isoform was somewhat attenuated in a mouse infection model, replicating more than 100-fold less at the inoculation site and failing to repress interleukin-6 and interferon-γ expression (74). As shown for VACV E3, mutation of key residues for dsRNA binding in OV20.0 strongly reduced interactions with PKR and diminished PKR inhibition (75). The E3 ortholog from myxoma virus, M029, which lacks the Zα domain, was also shown to inhibit PKR activation. A M029-deficient myxoma virus showed a strong replication defect in many tested cell lines, which was rescued after PKR depletion. This virus was also highly attenuated in a rabbit infection model (76). Comparative analyses of E3 orthologs from swinepox, myxoma, sheeppox, and Yaba monkey tumor viruses revealed that only swinepox and myxoma viruses could successfully rescue replication of an E3L-deficient VACV and prevent PKR activation. However, all of the tested viruses, except for an E3L revertant, showed strongly reduced pathogenicity in a mouse infection model (77). While this study demonstrated that not all E3 orthologs are efficient inhibitors of PKR, the attenuation of VACV expressing those proteins in mice could have been influenced by potential differences in expression kinetics, as E3L was driven by the native E3L promoter, whereas the other orthologs were driven by a synthetic early/late promoter, or protein function might have been impacted by the presence of a hemagglutinin (HA) tag (77). In agreement with the finding that the sheeppox virus E3L ortholog could not rescue VACV replication and PKR inhibition (77), the sheeppox ortholog was unable to inhibit sheep PKR in a transfection-based reporter assay (57). It is currently unknown why different E3 orthologs are more efficient PKR inhibitors than others, but it might be related to differences in dsRNA binding, PKR interaction, or preferential interaction with other proteins. Monkeypox virus contains an N-terminally truncated E3 homolog that uses the corresponding start codon as the vaccinia virus p20 isoform. VACV expressing only E3 p20 did not inhibit PKR as efficiently as E3 p25, especially later in infection (≥9 hours), despite retaining dsRNA binding abilities (78, 79). This virus was able to replicate in most cell lines but was replication deficient in mouse JC cells. Similarly, VACV expressing the monkeypox virus E3L ortholog (F3L) instead of E3L replicated poorly in JC cells. However, monkeypox virus was able to replicate as well as the wild-type VACV, which indicated that monkeypox virus could compensate for the lack of the Zα domain with other mechanisms (79). A possible explanation is the reduced dsRNA formation as compared with VACV, as discussed below (80).

## INHIBITION OF PKR BY VACV K3

VACV K3 is a 10.5 kDa protein. It is homologous to the eIF2α kinase-interacting N-terminal S1 domain of eIF2α and acts as a pseudosubstrate inhibitor of PKR (81, 82). K3L-deficient VACV showed a slight replication defect in mouse L929 cells, which was exacerbated after interferon-α/β treatment, and showed strongly impaired synthesis of viral proteins (81). K3 was further shown to reverse PKR-mediated reporter gene expression and to suppress PKR and eIF2α phosphorylation (83). K3 was found to interact with the kinase domain of PKR and bind at the same region as eIF2α (84–87). The crystal structure of K3 showed a similar topology as the S1 receptor binding domain, a protein domain first found in ribosomal protein S1, associated with RNA binding proteins (82, 88). Using recombinant proteins, it was further shown that K3 strongly inhibited eIF2α phosphorylation and, to a lesser degree, PKR autophosphorylation, and that K3 interacted with PKR dimers rather than monomers (82). In a mouse infection model, a K3L-deleted virus showed an interesting dissemination defect after intratracheal infection, where a high viral load accumulated in the lungs, causing severe respiratory distress (89). In a yeast-based assay, K3 partially inhibited the toxic effects of human PKR and therefore incompletely inhibited human PKR. Further experiments using a yeast screen with randomly mutated K3L identified a hyperactive K3 variant (K3L-H47R) that inhibited human PKR considerably better. Interestingly, the corresponding position of the K3 mutation in eIF2α is directly adjacent to the phosphorylation site (S51), where an arginine is also found at this position (87). Additional studies focusing on the host range function of K3L will be discussed below.

In addition to PKR, VACV K3 was also shown to interact with the eIF2α kinases GCN2 and HRI in yeast and *in vitro* assays (87, 90, 91). The physiological importance of these interactions is currently not clear. The finding that the kinase domains of GCN2 and HRI have been under purifying selection during vertebrate evolution, as opposed to the kinase domain of PKR, which has been under positive selection (23), indicates that host-virus arms races did not substantially impact the evolution of the GCN2 and HRI kinase domains.

## K3 ORTHOLOGS IN OTHER POXVIRUSES

K3L orthologs are present in most orthopoxviruses, centapoxviruses, and clade II poxviruses, and thus evolved later than E3L (Fig. 2, K3 clade). In monkeypox and ectromelia viruses, the K3L ortholog is inactivated by unique, virus-specific point mutations and a deletion of 25 bp (ectromelia and monkeypox virus clade I), or 28 bp (monkeypox virus clade II), that include nucleotides encoding the conserved PKR-recognition motif KGYID (65). Whereas a K3L ortholog is present in Cotia virus (ORF 29), it is absent in its closest relative BeAn 58058 , due to a 9.6 kb deletion corresponding to Cotia virus ORFs 20 through 29 (92). The eptesipox virus K3 ortholog (ORF 012) contains a unique 86 aa C-terminal extension that does not contain discernible homology to other proteins. In a yeast growth assay, this K3 ortholog inhibited PKR from several bat species, and deletion of the C-terminal extension resulted in a loss of PKR inhibition of some of the tested bat PKRs, indicating that this extension might increase the binding affinity (25). The nuclear magnetic resonance structure of M156, the K3L ortholog from myxoma virus, showed a similar structure as VACV K3. However, in contrast to K3, no suppression of human PKR-induced growth inhibition was observed in yeast assays (93). Positive selection has also been shown for the K3L orthologs in orthopoxviruses, which indicated that the evolution of K3L orthologs counteracts adaptive changes in PKR (24) or that K3L evolution was accelerated after host changes (94). Experiments with several other poxvirus K3 orthologs that affected PKR species-specificity are described below.

## HOST RANGE FUNCTION OF POXVIRUS E3 AND K3 ORTHOLOGS

E3L and K3L were shown to influence the cell line tropism by using E3L- or K3L-deleted viruses. An E3L-deleted virus was able to replicate in European rabbit RK13 cells and Syrian hamster BHK cells, but not in human HeLa cells. In contrast, a K3L-deficient VACV showed a replication defect in BHK-21 cells, while it could still replicate in HeLa, RK13 cells, and mouse L929 cells (52, 53, 62, 81). The presence of both PKR inhibitors in VACV indicated that they evolved and were maintained to expand the viral host range. As a possible explanation for the host range function of E3 and K3, different PKR and dsRNA levels in the tested cell lines were discussed (62). The discoveries that PKR evolved under intense positive selection and diversified in multiple vertebrate lineages indicated PKR species-specific differences in the susceptibility to viral inhibitors as an explanation for the host range function of PKR inhibitors. This explanation was more apparent for the case of K3, as it directly competes with eIF2α for binding to PKR, and a clustering of positively selected residues in PKR was identified that are adjacent to or coincide with the putative eIF2α/K3 interaction areas. Importantly, yeast assays revealed that PKRs from different primate species had differential sensitivity to K3 inhibition and that the substitution of positively selected residues in human PKR with residues from other species resulted in changes in the sensitivity to K3 inhibition (23, 24). Transfection-based reporter assays with HeLa PKR-knockdown cells showed that human PKR was only weakly inhibited by VACV K3, whereas mouse PKR was strongly inhibited. Substitution of alanine 488 in human PKR with a glutamic acid, which is found in mouse PKR at the corresponding position, made PKR more sensitive to K3 inhibition, whereas the corresponding substitution in mouse PKR made it more resistant (23). Subsequent work showed that European rabbit and Syrian hamster BHK cells were also sensitive to K3 inhibition (58) and that replication of a VACV strain deficient in both E3L and K3L could be efficiently rescued by the expression of K3L from a different locus in BHK-21, mouse 3T3, and RK13 cells (59), further strengthening the correlation of PKR inhibition and VACV replication.

Studies to uncover the molecular basis for the host range function of E3L revealed that Syrian hamster PKR was resistant to E3 inhibition while being sensitive to K3 (26). This was shown in yeast, cell line reporter, and infection assays. PKR of four different hamster species showed differential sensitivity to K3 and E3 inhibition. Both Syrian and Turkish hamster PKRs were resistant to E3 inhibition, whereas Chinese and Armenian hamster PKRs were sensitive. Syrian, Turkish, and Chinese hamster PKRs were sensitive to K3 inhibition, whereas Armenian hamster PKR was resistant. Human PKR showed similar sensitivities as Armenian hamster PKR (sensitive to E3 inhibition, but not weakly inhibited by K3), whereas mouse PKR showed similar sensitivities as Chinese hamster PKR (sensitive to E3 and K3 inhibition). In agreement with these findings, K3L but not E3L was dispensable in Armenian hamster PKR-expressing cells, whereas the opposite was observed in Syrian hamster PKR-expressing cells. A Syrian hamster PKR mutant containing two amino acid residues from Armenian hamster PKR in helix αG conferred resistance to wild-type VACV. These findings established that the host range functions of E3L and K3L are due to species-specific sensitivities of PKRs.

In order to be identified as host range genes in this system, PKR of a given species has to show opposing sensitivities to E3 and K3, as is the case for human and Syrian hamster PKRs. Mutational analyses revealed that two residues in Armenian hamster PKR that have been under positive selection in rodents conferred resistance to K3 inhibition, and introduction of these residues into Syrian hamster PKR made it resistant to both E3 and K3 inhibition. Importantly, a cell line expressing this hybrid PKR as its sole PKR inhibitor was resistant to infection with the wild-type VACV. The resistance of Syrian hamster PKR to E3 inhibition was more complex, and no single residues were identified that confer this resistance. Instead, the regions linking the dsRBDs to the kinase domains were shown to play an important role in the sensitivities to E3 inhibition between Syrian and Armenian hamster PKRs. The resistance of Syrian and Turkish hamster PKRs to E3 is not compatible with the dsRNA sequestration model as the major mode of E3’s action. Instead, it supports a model in which E3 forms heterodimers with PKR, preventing functional PKR homodimerization. Resistant PKR could either withstand interaction with E3, or PKR could still adopt functional dimerization and proper orientation of the kinase domains, possibly aided by the linker region, even when E3 is bound to the dsRNA-PKR complex (26).

The impact of host species-specific PKR inhibition was also investigated for K3L orthologs from other poxviruses. Comparative analyses of four orthopoxvirus K3 orthologs, which share between 81% and 99% aa identity with one another, showed surprisingly different inhibition profiles of PKRs from 17 mammals. While the variola virus K3 ortholog showed a unique inhibition profile, it inhibited human PKR only slightly better than VACV K3. In contrast, the tested cowpox and camelpox K3 orthologs inhibited human PKR much more strongly. While some PKRs (pig and guinea pig PKRs) were resistant against all four tested K3 orthologs, other PKRs (horse and dog PKRs) were very sensitive to all. The inhibition correlated well with replication of VACV that expressed either VACV or camelpox K3, with the latter virus replicating ~5,000-fold better than the former in HeLa cells. In contrast, both viruses replicated comparably well in bovine cells, whose PKR was inhibited strongly by either VACV or camelpox K3 (58).

**Fig 3:**
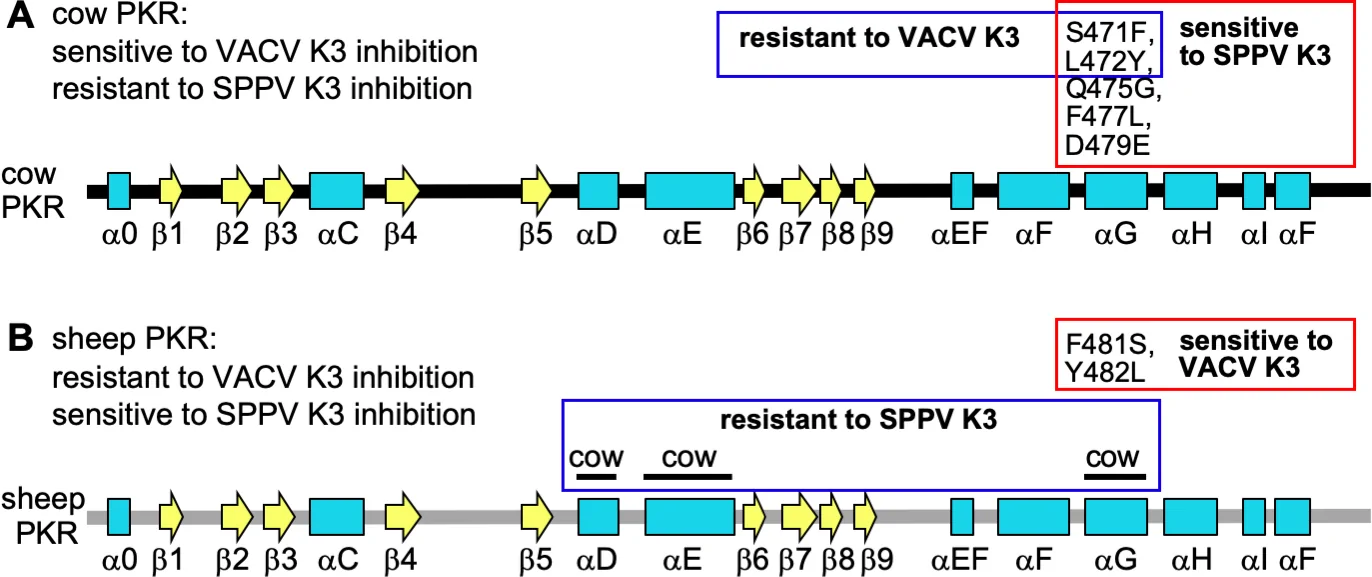
Residues in cow and sheep PKR that determine sensitivity to VACV and SPPV K3 inhibition. Schematic representation of cow and sheep PKR. The secondary structure elements are based on the structure of human PKR (95). (**A**) Amino acid residues in cow PKR that determine sensitivity to VACV K3 inhibition, or resistance to SPPV K3, are marked by a blue or red rectangle, respectively. (**B**) Amino acid residues in sheep PKR that determine resistance to VACV K3 inhibition are marked by a red rectangle. Regions in sheep PKR that determine sensitivity to SPPV K3 are indicated by bold lines and marked with a blue rectangle.

Surprising differences in PKR sensitivity were also observed for capripoxvirus K3 orthologs, from sheeppox and goatpox viruses, which infect both sheep and goats, and lumpy skin disease virus, which infects cattle. All three K3 orthologs strongly inhibited sheep and goat PKRs but weakly inhibited cow PKR. Human PKR showed comparable sensitivity as sheep and goat PKRs, whereas mouse PKR showed comparable sensitivity to cow PKR. Interestingly, the sensitivity to VACV K3 inhibition of the tested PKRs was inversed. This also correlated with replication of VACV expressing these K3 orthologs, with VACV expressing sheeppox K3 replicating to high titers in sheep, goat, and human cells, but to low titers in cow cells (57). In a follow-up study with 20 mammalian PKRs, the same contrasting sensitivity patterns to VACV and sheeppox virus K3 inhibition were observed for some PKRs. In contrast, some PKRs were either sensitive or resistant to both K3 orthologs (27). Mutational analyses showed that the swap of two residues (S471F, L472Y) in helix αG of cow PKR with the corresponding residues in sheep PKR, and the corresponding changes in sheep PKR (F481S, Y482L), completely reversed sensitivities to VACV K3, but only partially to sheeppox virus K3 (Fig. 3A and B). Swapping of additional residues in helix αG was necessary to make cow PKR fully sensitive to sheeppox virus K3 inhibition (Fig. 3A). In contrast, swapping of cow helix αG into sheep PKR made it only partially resistant against sheeppox virus K3. Attaining full resistance required the additional replacement of helix αD and/or αE (Fig. 3B) (27). This indicates that different regions in PKR differentially contribute to sensitivity to different K3 orthologs. While it is likely that PKR helix αG is the main interactor with K3 orthologs, as inferred from the co-crystal structure of PKR with eIF2α (95), residues in helix αD and αE were previously shown to modify sensitivity to K3 orthologs, presumably by modifying the positioning of helix αG (23, 24, 96).

Additional evidence that PKR acts as an important host range factor and that viral K3 orthologs function to overcome this barrier comes from studies of the interaction between primate PKRs and yatapoxvirus K3 orthologs (60). K3 orthologs from Tanapox virus (TPV 012) and Yaba monkey tumor virus (YMTV 012) share 75% aa identity. Despite both K3 orthologs being closely related, they showed different PKR inhibition profiles, in which TPV 012 was more broadly effective than YMTV 012 in inhibiting the activity of PKR from 15 primate species, which was tested in a luciferase-based assay. The key findings of the study are that the yatapoxvirus K3 orthologs inhibited PKR in a species-specific manner. Whereas TPV 012 inhibited 11 PKRs, including human PKR, more effectively than YMTV 012, both yatapoxvirus K3 orthologs inhibited four PKRs from other primates comparably well. Hybrid experiments swapping regions between TPV 012 and YMTV 012 showed that the C-terminal region was the major determinant of differential PKR inhibition. Furthermore, E3L/K3L-deficient VACV expressing TPV 012 replicated more effectively in primate-derived cell lines (~1,000-fold in BS-C-40, ~100-fold in Pro1190, and ~10-fold in Gibbon Tert cells) than VACV expressing YMTV 012, supporting the idea that PKR inhibition by yatapoxvirus K3 orthologs can influence host cell specificity and viral replication (60).

Myxoma virus (MYXV) infection is restricted to rabbits and hares and is mostly mechanically transmitted by biting arthropods, such as mosquitoes and fleas. Whereas MYXV causes only mild but long-lasting localized infections in its natural brush rabbit (*Sylvilagus bachmani*) and American tapeti (*Sylvilagus brasiliensis*) hosts, it is highly virulent in European rabbits (*Oryctolagus cuniculus*), causing fatality rates of ~99% (97). The attenuation of MYXV after the deliberate release in Australia and Europe, and the concomitant increased resistance of European rabbits, represents one of the best-studied examples of virus-host coevolution at the population level (98). The MYXV K3 ortholog M156 was found to specifically inhibit PKR from rabbits but not PKR from other mammals (13, 99). Importantly, an M156 variant (M156R-L98P) from evolved Australian MYXV isolates (100, 101) showed a loss of function, and resulted in strongly reduced MYXV replication in rabbit cells, and might thus contribute to the attenuation of MYXV in Australia (99).

## THE BUNNY PARADOX, OR CONSEQUENCES OF PARTIAL PKR INHIBITION

The species-specific inhibition of PKR by poxvirus proteins can be explained by molecular arms races between hosts and viruses, which resulted in the positive selection of PKR variants with increased resistance to viral inhibitors, and adaptations of the viral inhibitors to inhibit PKR of host species more efficiently (23, 24, 102). In cases in which viral inhibitors do not inhibit PKR, this represents an efficient barrier for poxvirus replication. Because PKR has potent antipoxviral activity, we initially hypothesized that efficient PKR inhibition would correlate with the virulence of myxoma virus in rabbit hosts. However, PKR from the natural brush rabbit host was inhibited much more strongly than European rabbit PKR (13, 14). This is the opposite of the expectation that more complete PKR inhibition should favor viral replication and virulence. A possible explanation for this paradox lies in the consequences of partial PKR inhibition and that efficient virus replication is not the sole contributor to virulence. As detailed in the PKR pathway section, PKR activation can result in the activation of the NF-κB pathway, mediated by the rapid turnover of its inhibitor IκB. However, when PKR is highly active, translation is strongly inhibited, and thus NF-κB-induced mRNAs are not effectively translated. We hypothesized that full PKR inhibition would prevent NF-κB activation, whereas partial PKR inhibition would lead to intermediate NF-κB activation, transcription of pro-inflammatory cytokine genes, and also permit intermediate translation of some NF-κB target mRNAs. Indeed, co-transfection of PKR-deficient HeLa cells with European rabbit PKR and M156R resulted in transcription of NF-κB target genes tumor necrosis factor α and interleukin-6, whereas no induction was observed when brush rabbit PKR was co-transfected with M156R. Consistently, expression of an NF-κB reporter was induced when European rabbit PKR and M156 were co-expressed but was repressed when brush rabbit PKR and M156 were co-expressed (13). Swapping four aa residues in helix αG between European rabbit and brush rabbit PKR reversed the susceptibilities to M156 inhibition and NF-κB reporter expression (14). Congruently, a M156 variant that was identified in a yeast screen that inhibited European rabbit PKR but not brush rabbit PKR better conferred strongly reduced NF-κB reporter expression when European rabbit and M156 were co-expressed (13). We speculate that the intermediate PKR inhibition and associated functional NF-κB activation might cause increased leukocyte migration and MYXV virulence, which have to be analyzed in follow-up studies. These studies also offer a new molecular perspective on host-virus arms races. Better inhibition of antiviral pathways might not necessarily mean increased virulence. In the case of brush rabbit PKR, evolving complete resistance against M156 may not be possible because a single or a few mutations at the PKR-M156 interface can result in intermediate inhibition, which could possibly lead to higher MYXV virulence. Instead, it might be better for the host to forfeit the arms race and tolerate strong PKR inhibition to keep the infection localized. Relatedly, M156 might have evolved to inhibit PKR of the natural MYXV hosts more efficiently because its transmission might benefit from a long-lasting local infection without extensive harm to its host.

## NILE CROCODILEPOX VIRUS 157

The genus *Crocodylidpoxvirus* includes two species: Nile crocodilepox virus (CRV) and saltwater crocodilepox virus. Their genomes lack E3L and K3L orthologs (103–105). However, database and comparative protein analyses revealed a putative dsRNA binding protein encoded by CRV 157 that is only distantly related to poxvirus E3 orthologs. It contains a dsRBD at the N-terminus and a unique domain at the C-terminus (106). Subsequently, CRV 157 was shown to have the highest sequence identity with a dsRBD from *Streptomyces gossypiisoli* ribonuclease III that extends into the C-terminal part, bind to poly(I:C), co-localize with chicken PKR, and inhibit PKR from various species in reporter assays. Expression of the dsRBD of CRV 157 alone showed reduced PKR inhibition, indicating a function for the C-terminus. Furthermore, CRV 157 was able to rescue replication of a PKR-inhibitor-deficient VACV in rabbit RK13 cells and to inhibit eIF2α phosphorylation. Replication of VACV + CRV157 was delayed compared with VACV + E3L but ultimately reached comparable titers after 72 h (107). A CRV 157 ortholog (ORF 198) is found in saltwater crocodilepox virus that shows 86% aa sequence identity to CRV 157.

## CReP/GADD34 HOMOLOGS

Canarypox virus contains a homolog of the PP1 interaction proteins CReP/GADD34 called CNPV231 (108). Homology is largely restricted to a 53 aa fragment that contains both the PP1- and eIF2α-binding motifs (109). Sequence identity in this part is higher between CNPV231 and chicken CReP (58.5%) than between CNPV231 and chicken GADD34 (39.6%). . We therefore consider it most likely that an ancestor of canarypox virus acquired a CReP gene from a host. Pull-down assays in yeast showed that CNPV231 interacted with eIF2α, rescued yeast from the toxic effects of human PKR expression, and reduced both PKR and GCN2-induced eIF2α phosphorylation (109). Additional experiments are required to substantiate the role of CNPV231 to inhibit the PKR pathway, particularly in the context of infection. Among chordopoxviruses, CNPV231 orthologs are present in all clade B avipoxviruses but not in other avipoxviruses (Fig. 2).

## DECAPPING ENZYMES D9 AND D10 REDUCE dsRNA ACCUMULATION

VACV encodes two decapping enzymes named D9 and D10, encoded by OPG121 and OPG122, respectively, which remove the 5′ terminal 7-methylguanosine (m7G) cap from both cellular and viral mRNA by hydrolysis of the phosphodiester bond, leaving an exposed monophosphate at the 5′ end of the mRNA (110–112). These two enzymes are structurally similar to other eukaryotic Nudix hydrolases, such as Dcp2 (113). The enzymatic activity of decapping enzymes is mediated by the Nudix/MutT motif that hydrolyzes nucleoside diphosphate. The Nudix motifs are highly conserved and often located in the central regions of the decapping enzymes (114). D9 and D10 show 25% sequence identity with one another and are expressed at different stages in the replication cycle, with D9 being expressed early and D10 being expressed at intermediate and late stages of infection (51, 110, 111). The decapping of mRNAs reduces the translation initiation efficiency and thus contributes to the shutoff of host protein translation, promoting therefore viral protein synthesis. The differential temporal expression of D9 and D10 significantly contributes to a more precise regulation of mRNA turnover-mediated pathways (115). While both D9 and D10 act as negative regulators of gene expression through mRNA decay mechanisms and control of RNA abundance during infection, they display distinct roles. While D9 mainly targets viral transcripts for decay (116), D10 represents a constitutively broad-acting decapping enzyme, depleting preferentially transcripts derived from intron-containing genes than from intronless genes (112). Inactivation of D9R had no effect on VACV replication, in contrast to inactivation of D10R (110, 111). However, simultaneous inactivation of both genes induces a profound host restriction of viral replication, suggesting complementing functions of D9 and D10 (117). D9 and D10 also display differences in substrate recognition, with D10 showing stronger decapping activity (111). Decapped mRNAs are rapidly degraded by the host 5′−3′ exoribonuclease XRN-1 (117–119). Post-replicative poxvirus mRNAs contain unique mRNA 5′-poly(A) leaders, which can counteract the negative effects of this decapping activity on the translation of viral mRNA (120). D9 and D10 promote, therefore, a selective translation of viral transcripts with 5′-poly(A) leaders (121).

Another important function of D9 and D10 is the strong reduction in the amount of dsRNA, as they both act on single-stranded and dsRNA (122) by decapping dsRNA that is subsequently degraded in an XRN-1-dependent manner. This reduces the activation of dsRNA-sensing antiviral pathways, especially PKR and OAS/RNase L (56, 117, 118). Infection with a VACV strain containing mutations in the catalytic sites of both D9 and D10 led to the accumulation of dsRNA, which correlated with an increase in PKR and eIF2α phosphorylation and RNA degradation, as well as with strongly reduced virus replication in African green monkey BS-C-1 cells. Additionally, greatly decreased virus replication was also observed in HeLa cells, as well as mouse embryonic fibroblasts infected with D9/D10 catalytic site mutants. Interestingly, replication of the D9/D10 mutant was only slightly affected in BHK-21 cells (117). A possible explanation for this host range function is that the amounts of PKR in BHK-21 cells are quite low, as evidenced by partial replication of VACV lacking both E3L and K3L and a strong reduction in replication of a K3L-deficient VACV after interferon treatment, which was reversible after PKR depletion (26). Infections of BALB/c mice with either single or double mutated D9 and D10 VACV showed complete attenuation when the mice were infected with 10^5^ and 10^6^ PFU. D9 mutated VACV was only slightly attenuated compared with the wild-type virus, as indicated by the finding that three out of five or five out of five mice met euthanasia criteria after infection with 10^5^ or 10^6^ PFU, respectively. In contrast, infection with D10 mutated VACV caused no weight loss at 10^5^ PFU and strong weight loss but full recovery at 10^6^ PFU. These data showed that both D9 and D10 are virulence factors but differentially affected disease outcome (117). While VACV D9 and D10 prevent excess PKR activation, they cannot compensate for the loss of both E3 and K3 during vaccinia virus infection, as indicated by the inability of VACV lacking both inhibitors to replicate in the following cell lines: HeLa and 293 (both human), Tert-GF (gibbon), BSC-40, Pro1190, and CV-1 (all African green monkey), OA1 and OA3 (sheep), CSM (goat), BT (cow), PK15 (pig), 3T3 (mouse), Don, and V79-4 (both Chinese hamster), and AHL-1 (Armenian hamster). The only exception is BHK-21 cells, in which a little replication was observed, likely due to the aforementioned hypothesized low PKR expression levels, and cells in which PKR was deleted or depleted, or that overexpress viral PKR inhibitors (E3 and/or K3) (26, 57–59, 123–125).

## D9/D10 HOMOLOGS IN OTHER POXVIRUSES

**Fig 4:**
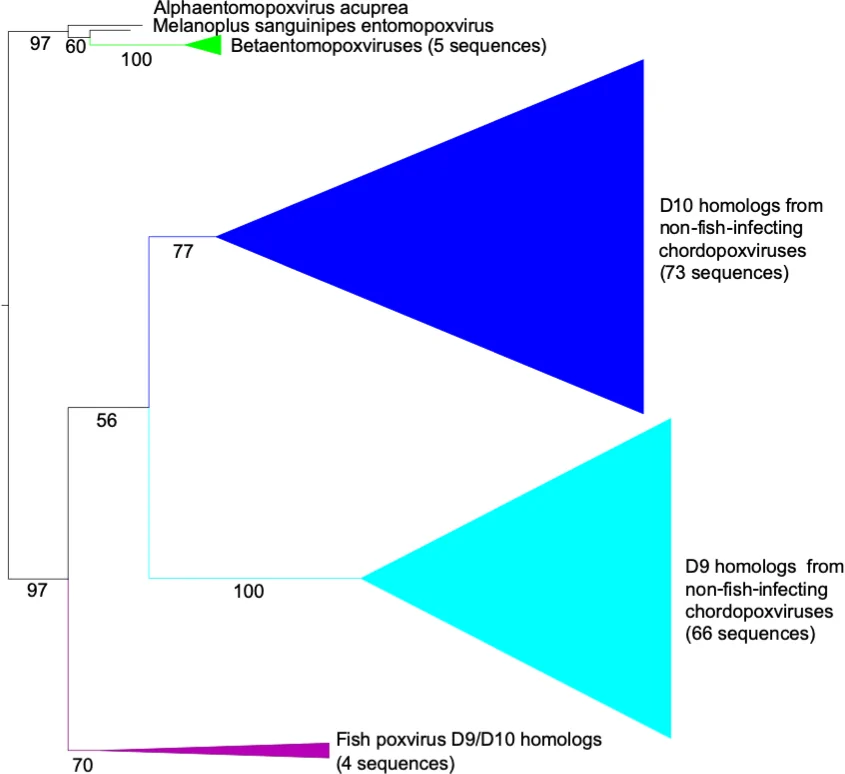
Phylogeny of poxvirus D9/D10 homologs. A multiple sequence alignment was created from 150 chordopoxvirus D9/D10 homologs using MUSCLE (70). Poorly aligned fragments were removed using Gblocks 0.91b (71). A maximum likelihood phylogenetic tree was generated with PhyML 3.0 using 100 bootstrap replicates (72) and visualized in FigTree v1.4.3 (http://tree.bio.ed.ac.uk/software/figtree/). The resulting phylogenetic tree was rooted to entomopoxvirus D9/D10 homologs. Bootstrap support is indicated below the branches. The branches of major clades were collapsed and are shown as triangles for better clarity.

Our survey for D9/D10 orthologs revealed that all completely sequenced insect-infecting entomopoxviruses (not included in Fig. 2), as well as four fish-infecting poxviruses, contain only one D9/D10 homolog (Fig. 2). Entomopoxviruses and fish poxviruses lack any other recognizable eIF2α kinase pathway inhibitors. A phylogenetic analysis (excluding the BeAn 58058 homologs because of truncations in those proteins) showed that the fish D9/D10 homologs formed a sister clade to the other chordopoxvirus D9 and D10 homologs, the latter of which formed two well-supported clades (Fig. 4). This indicates that an ancestral decapping enzyme-encoding gene duplicated early in the evolution of chordopoxviruses, after the split from the fish poxvirus lineage. Salmon gill and Japanese seabream poxviruses D9/10 homologs contain an in-frame deletion encoding approximately 48 aa, relative to the other D9/D10 homologs. This deletion covers most of the insertion domains found in VACV D9 and D10, which are absent from other decapping enzymes, such as the cellular decapping enzyme Dcp2 (122). Carp edema virus and *Plecoglossus altivelis* poxvirus contain an approximately 80-aa long C-terminal extension that is absent from other poxvirus D9/10 homologs. All other surveyed chordopoxviruses contain full-length D10 homologs, with the exception of BeAn 58058 , whose D10 ortholog shows a 62-aa truncation at the C-terminus, relative to the Cotia virus ortholog, which corresponds to aa 187 to 248 in VACV D10. This truncation is caused by a 1 bp insertion, which results in a frame shift causing a premature stop codon. Most poxviruses also contain a D9 homolog, with the exception of parapoxviruses and Wadden Sea poxvirus, in which no D9 ortholog could be identified. The BeAn 58058 D9 ortholog contains a 24-aa truncation at the N-terminus, relative to the Cotia virus ortholog, due to a 1 bp insertion at nucleotide position 69, in respect to the start codon in Cotia virus.

Only a few studies investigated the function of D9/D10 homologs in other poxviruses. OV71 is a D10 ortholog and the only decapping enzyme in orf virus (ORFV). OV71 was shown to be expressed early during ORFV replication and accumulated to higher levels late in replication. OV71 was able to bind both ssRNA and dsRNA. ORFV strains in which OV71 was inactivated by point mutations or a deletion showed strongly reduced replication in primary goat fibroblast (FB) and 293T cells, which correlated with low levels of viral but not cellular mRNA (115). A lumpy skin disease virus (LSDV), in which the D10 homolog LSDV 087 was deleted, showed a weak replication defect (about 2-fold) in bovine MDBK cells. Surprisingly, LSDV 087 was identified as an activator of the cGAS/STING pathway by interacting with and inhibiting the degradation of STING. This activity was largely independent of the catalytic activity of LSDV 087, whereas VACV D10 did not have this effect (126). In agreement with the studies on VACV D10 (112), LSDV 087 was found to predominantly lead to the degradation of mRNAs generated from intron-containing but not intronless genes (126).

## OTHER PUTATIVE dsRNA-REDUCING POXVIRUS PROTEINS

Comparative analyses of dsRNA accumulation between VACV and monkeypox virus or VACV and ectromelia virus showed that more dsRNA was detected in cells infected with VACV than with monkeypox virus or ectromelia virus, indicating that poxviruses can use other strategies to counteract dsRNA accumulation (80, 127). This indicates that some poxviruses might have evolved different strategies to prevent dsRNA accumulation. Interestingly, both monkeypox and ectromelia viruses lack K3L orthologs.

One VACV protein that has been implicated in reducing dsRNA accumulation is K1 because more dsRNA was detected by an antibody in a K1L (OPG039)-deleted virus, which coincided with elevated eIF2α phosphorylation levels. This virus also showed other phenotypes, including strongly reduced E3 expression (128). It is possible that the decreased E3 expression resulted in higher dsRNA detection because more dsRNA epitopes, which are usually masked by E3, would be available for antibody detection. Additionally, the lower E3 expression could also explain activation of PKR. Transiently expressed K1 was not able to enhance translation and thus is likely not directly involved in inhibiting PKR (83). Moreover, E3L- and K3L-deleted VACV was unable to replicate in most cell lines tested despite the presence of K1L (26, 57–59, 123–125). K1 is also an ankyrin repeat-containing protein and was identified as a host range protein because its absence severely affected virus replication in various human cell lines and RK13 cells, but less so in mouse L and DBT, Vero, and BHK cells. K1L orthologs are found in centapoxviruses and most orthopoxviruses, with the exception of taterapox, camelpox, and modern variola viruses. Although these three host-restricted viruses are closely related and belong to the same clade, their K1L orthologs are disrupted by independently acquired unique gene-inactivating mutations (65). Interestingly, intact K1L orthologs were identified in two ancient variola virus genomes from Scandinavia that date between 603 and 770 CE during the Viking Age (129).

Another interesting candidate for reducing dsRNA levels is A24, a subunit of the RNA polymerase. A mutation in A24 (A24R, OPG151) was identified in an isatin-beta-thiosemicarbazone (IBT)-resistant VACV strain. IBT leads to increased read-through transcription from convergent transcripts and induces the production of excess dsRNA, which in turn activated PKR and OAS/RNase L. The identified mutation caused an elongation defect and reduced the ability of the RNA polymerase to resume elongation from a paused state. This results in the production of less dsRNA (130, 131). In addition, other mutations in A24 and other RNA polymerase subunits were identified in IBT-resistant VACV (132). Importantly, adaptive mutations in A24 were also identified during serial passage of attenuated VACV lacking E3L (described in more detail below) (124, 125, 133, 134). In addition to A24R, mutations in other genes such as DNA polymerase, other RNA polymerase-encoding genes, and several other genes have been identified in these studies, indicating that the lack of efficient PKR inhibitors can be compensated by different mechanisms, the nature of which still needs to be determined.

## PKR INHIBITOR-DEFICIENT POXVIRUSES AS TOOLS FOR EVOLUTIONARY STUDIES AND FOR EFFICIENT SELECTION OF RECOMBINANTS

### Gene amplifications in poxviruses

The requirement for PKR inhibitors for VACV replication provides opportunities to use PKR inhibitor-deficient VACV strains to elucidate fundamental processes in viruses. We have already discussed how the research on PKR inhibitors advances our understanding of virus host range. Gene amplification is an evolutionary mechanism that provides fitness gains in all life forms, including viruses (135). A naturally evolved gene amplification in poxviruses can be observed for some poxvirus virulence and host range genes, e.g., the C7L (OPG027) family genes of leporipoxviruses, in which three closely related genes (062, 063, and 064) are found in tandem (136). Gene amplification in experimental settings in poxviruses was first reported after the small ribonucleotide reductase gene locus (F4L; OPG048) was amplified in VACV that was selected in medium containing the ribonucleotide reductase inhibitor hydroxurea (137). To study the mechanisms of how viruses can adapt to overcome the antiviral host response, serial passaging of an E3L-deficient VACV strain in HeLa cells was employed. This virus contains K3 as the sole inhibitor, which, as discussed above, is a weak inhibitor of human PKR but still provides enough PKR inhibition to allow limited virus replication. After about six passages, approximately 10-fold increased virus replication was observed, which coincided with an amplification of the K3L locus (138). Additionally, variations in some genes were observed, including one in K3L, which led to an H47R substitution, the same aa substitution that was previously observed in a yeast mutagenesis screen (87), described in the VACV K3L section. VACV containing a single copy of K3L-H47R replicated significantly better in HeLa cells than the parental virus. After passaging in Syrian hamster BHK cells, whose PKR is effectively inhibited by K3, the K3L locus amplification collapsed into a single or only a few copies, leading the authors to propose an accordion model in which gene amplifications provide a temporary fitness advantage that allows for virus replication and the selection of other beneficial mutations, which can be followed by the collapse of the amplified locus (138). This model was later supported by passaging an E3L- and K3L-deficient VACV, in which a PKR inhibitor from the rhesus cytomegalovirus PKR antagonist (RhTRS1) was inserted into the E3L locus in African green monkey cells. Here, amplification of the rhTRS1 gene was observed, which correlated with a strong increase in virus replication (124). Surprisingly, further investigations did not detect mutations in RhTRS1 but in A24R and A35R (OPG163), which by themselves increased replication of the parental virus (133). When the African green monkey cell-adapted virus was passaged in human fibroblasts, an approximately 100-fold better replication was observed after nine passages. Interestingly, some single nucleotide polymorphisms (SNPs) in the parental populations decreased in frequency, whereas other SNPs and short indels strongly increased in frequency in the passaged virus populations. Strikingly, many of these variations affected viral RNA polymerase subunits (124, 125, 133, 134). Conclusions from these studies are that duplications of poxvirus genes occur frequently, but most of the time are not selected for because they do not confer selective advantages (124, 138). Only under evolutionary pressures, such as after host switches, might these amplifications provide advantages to act as molecular footholds that allow the accumulation of adaptive variations, facilitating host changes (124, 125).

### Molecular mechanisms of host-gene capture

A high number of genes in large DNA viruses were originally acquired from hosts through horizontal gene transfer (HGT). Many of the encoded proteins are involved in modulating the host immune response (34, 139–143). Two recent complementary studies used PKR inhibitor-deficient VACV to elucidate the mechanisms of how poxviruses can acquire host-derived genes (144, 145). To allow VACV replication and to distinguish between DNA- and RNA-mediated gene transfer mechanisms, RK13 cells were stably transfected with either a mCherry-E3L or mCherry-K3L fusion gene that contains a poxvirus early/late promoter and an intron. Progeny viruses were subsequently screened on PKR-competent RK13 or BHK cells, respectively, allowing only viruses to replicate that acquired either E3L or K3L. Aided by mCherry expression, both studies identified gene transfer events, which were characterized by spliced-out introns, untemplated 3′ poly(A) tails, and target site duplications of ~15 bp surrounding the integration sites. These signatures are hallmarks of long interspersed nuclear element-1 (LINE-1)-mediated retrotransposition. While the gene transfer of these specific transcripts was very rare and occurred in ~1 in 23 million to 1 in 50 million plaque-forming virions, the actual transfer frequency of host mRNAs is likely much higher, given that the E3L and K3L transcripts make up only a small fraction of total cellular mRNA (144, 145). One discrepancy between both studies was the location of integration sites. Whereas mCherry-K3L was found in 9 out of 10 cases towards the terminal genome regions, the 20 mCherry-E3L integrations were distributed throughout the genome and in 8 cases led to the disruption of essential genes. In the latter cases, co-infections with the parental virus were identified, in which the parental viruses provided the essential gene, and the HGT viruses provided E3L. Serial passaging led to the recombination of both replication-deficient viruses into single replication-competent viruses via non-homologous recombination (144). An interesting observation was that in one case, mCherry-E3L integrated together with a host-derived polyadenylated U2 small nuclear RNA, indicating that two gene products could integrate together during HGT (144). While these landmark studies revealed that poxviruses can acquire host genes via RNA-mediated mechanisms, a limitation of this work is that in both cases, the used viral genes were optimized during virus evolution. In order to investigate if a host gene could provide replication benefits, Fixsen et al. inserted human eIF2α into the VACV-lacking E3L and K3L, which led to slightly improved virus replication. Over three passages, an ~10-fold better virus replication was observed, which coincided with amplification of the integrated genes. However, virus replication was not sustained beyond four passages (145). Open questions remain on which host genes can provide durable virus fitness advantages and whether parts of host genes can function as poxvirus promoters. Moreover, the recombination between two deficient VACV can serve as a valuable model to study non-homologous recombination in poxviruses.

### PKR inhibitors as selection markers

Similar to their use as selection markers to probe horizontal gene transfer, E3L and K3L can be also used as potent selection markers to insert other genes into VACV, including foreign genes or PKR inhibitors from other poxviruses (146, 147). In a proof-of-principle study, human herpesvirus 8 (HHV8) glycoprotein K8.1 was inserted together with the taterapox virus K3L ortholog into the K3L locus of VACV lacking E3L and K3L. Selection was performed in BHK cells, in which the parental virus does not replicate efficiently (146). A similar strategy was used that allows the seamless integration of genes into the E3L locus of E3L/K3L-deficient VACV. Here, a gene of interest can be inserted into the E3L locus and will be transcribed from the native E3L promoter, which is followed by mCherry-E3L fusion gene that acts as a selection marker. The recombination vector contained ~500-nucleotide homology arms from the 5′ to 3′ regions of E3L, for integration into the E3L locus, and ~150 nucleotides with homology to the 3′ region of E3L located between the gene of interest and mCherry-E3L. The 3′ homology regions facilitate intramolecular recombination to remove the mCherry-E3L gene after successful selection and to allow the scarless integration of transgenes (147). Both methods have the advantage that they allow highly efficient selection of recombinants when the cells express PKR that is sensitive to the used PKR inhibitors.

## WHY DO SOME POXVIRUSES LACK KNOWN PKR PATHWAY INHIBITORS?

The work with VACV and myxoma virus lacking PKR inhibitors showed that they can only replicate in PKR-deficient or PKR-inhibitor-expressing cells, which indicates that a PKR inhibitor is necessary for replication of these viruses, despite encoding decapping enzymes. Similarly, the presence of independently evolved PKR pathway inhibitors in crocodylidpoxviruses and clade B avipoxviruses makes it likely that they inhibit PKR in their respective hosts, although more work is needed to address this. Because all poxviruses likely produce high amounts of dsRNA, an important question is how poxviruses that lack known PKR inhibitors counteract the effects of PKR activation. Characterized PKR pathway inhibitors are not found in fish poxviruses, macropoxviruses, clade A and C avipoxviruses, molluscipoxviruses, the related equine contagiosum-like virus, and *Rousettus* bat poxvirus (Fig. 2), which all infect hosts that possess PKRs. In an effort to identify PKR inhibitor candidates, a recent study used structural homology modeling to identify dsRBD-related proteins in molluscipoxviruses and macropoxviruses. These proteins contain additional predicted domains that are not present in E3 or CRV157 and have some but not all aa important for dsRNA-binding conserved (66). Future studies should reveal if those proteins are genuine dsRNA-BPs and whether they can inhibit PKR. Similarly, poxvirus inhibitors of fish PKZ have not been characterized. Although it seems likely that poxvirus E3 homologs might also inhibit PKZ, E3-containing poxviruses do not naturally infect fish. The finding that other DNA viruses, such as some fish-infecting iridoviruses and cyprinid herpesvirus 3, encode inhibitors of fish PKR and/or PKZ, indicates that they are important targets for fish-infecting viruses (148–150).

## CONCLUSIONS

Research on poxviruses, as detailed here for PKR inhibitors, has provided valuable mechanistic insights into how evolutionary conflicts between viruses and the vertebrate innate immune system shape viral host range, virulence, and the potential for cross-species transmission. The presence of various clade-specific poxvirus inhibitors highlights the multiple possibilities and trajectories of how poxviruses evolved different direct and indirect strategies to inhibit the PKR pathway. Recent studies further expanded this framework by showing that PKR viral antagonists not only antagonize the host translational shutoff pathway but also demonstrate that the extent of PKR inhibition can have additional effects, such as modulation of NF-κB-dependent inflammatory responses in a species-dependent manner (13, 14). The effects of this limited translation inhibition are an important concept that calls for further investigation and might also have important implications for other viruses. Overall, the work on poxvirus PKR inhibitors not only stimulated new conceptual models for a better understanding of viral immune evasion, host adaptation, and the molecular determinants of poxvirus host range but also provided important insights into other key evolutionary mechanisms, such as gene amplifications and the molecular basis of horizontal gene transfer. We expect that continued research will further enhance our understanding of virus evolution and modulation of the host response.
